# Sexually transmitted pathogens causing urethritis: A mini-review and proposal of a clinically based diagnostic and therapeutic algorithm

**DOI:** 10.3389/fmed.2022.931765

**Published:** 2022-08-26

**Authors:** Birgit Sadoghi, Birger Kränke, Peter Komericki, Georg Hutterer

**Affiliations:** ^1^Department of Dermatology and Venereology, Medical University of Graz, Graz, Austria; ^2^Department of Urology, Medical University of Graz, Graz, Austria

**Keywords:** urethritis, sexuall transmitted infections, *Chlamydia trachomatis*, Adenovirus, *Mycoplasma genitalium*, *Neisseria gonorrhoeae* (NG)

## Abstract

The purpose of this mini-review was to provide the latest information and concepts on diagnosis and treatment of the most common sexually transmitted pathogens causing urethritis. The incidence of several sexually transmitted infections that cause urethritis is increasing, and this genitourinary syndrome is among the most common reason young men see clinical care. The authors performed a literature search including the currently valid guidelines, and an overview of the most relevant pathogens is given. Moreover, the authors developed a clinically applicable diagnostic and therapeutic algorithm, because early diagnosis and correct treatment can sometimes prevent infected individuals from significant morbidity. Future research will focus on new methods to combat pathogens that cause urethritis, including vaccination.

## Introduction

Urethritis is defined as inflammation of the urethra. This syndrome is usually caused by sexually transmitted pathogens, including *Chlamydia trachomatis (CT), Neisseria gonorrhoeae (NG), Mycoplasma genitalium (MG*), and rarely pathogens such as Herpes simplex viruses 1 and 2, *Trichomonas vaginalis* (TV), or Adenovirus among some others (1–6). However, etiology can remain obscure in up to 50% of cases, which is then defined as idiopathic urethritis (6–8).

Clinical symptoms (=urethritis symptoms) include predominantly urethral discharge and dysuria, more rarely urethral irritation, urethral itch, or meatitis (1, 2, 4, 6, 9). Acute and persisting urethritis might result in significant morbidity, including arthritis, epididymo-orchitis, or prostatitis (6, 8).

Urethritis is verified either by:

(A) An increased number of polymorphonuclear leukocytes (PMNLs) in urethral exudate (also called urethritis signs) (4, 6).

Staining of the urethral smear can be performed with Gram stain or methylene blue (1). Microscopically, in most settings, diagnosis of urethritis is confirmed if ≥5 PMNL per high power (x1,000) microscopic field are present, of an average of 5 fields (1, 4, 9). Some authors, however, even chose higher numbers of PMNLs for diagnosis (high cut-off urethritis: ≥10 PMNLs) (10). This contrasts with recent recommendations of The Centers for Disease Control and Prevention (CDC) which recommends an even lower cut-off (2 PMNLs/HPF; regardless of the presence of symptoms) to define non-gonococcal urethritis (NGU) in men (6). This change was made because it was observed that lowering the cutoff to 2 PMNLs/HPF led to a significant increase in the detection of CT *via* Gram stain smear.

(B) Presence of causative agents *via* nucleic acid amplification tests (NAATs) of first void urine or urethral swab (4, 6).

Nucleic acid amplification tests are the gold standard for testing, not only as they allow non-invasive sampling, but even more due to their excellent specificity and sensitivity (11, 12). If urine is the material to be examined, not more than 20 ml of first void urine shall be used (1, 12, 13). As NAATs detect even non-viable organisms, a test of cures (TOCs) usually should not be performed before 3 weeks after completion of treatment (11).

(C) A positive leukocyte esterase test on first void urine or microscopy of sediment with more than 10 PMLS/HPF (6).

In the case of symptomatic urethritis, microscopy and NAATs should be performed. Clinicians should test for *Neisseria gonorrhoeae* (NG), *Chlamydia trachomatis* (CT), and *Mycoplasma genitalium* (MG) as a first step. If none of the above-mentioned pathogens is identified, other causative agents must be borne in mind, including Herpes simplex virus 1 and 2 (HSV –1, –2), Adenovirus, *Neisseria meningitidis* (NM), *Trichomonas vaginalis* (TV), *Haemophilus influenzae*, *Mycoplasma penetrans* (MP), *Treponema pallidum* (TP), or enteric bacteria among potential other infectious causes (6, 8, 12, 14).

Symptomatic NGU is usually managed syndromically and treatment is provided immediately. Contact tracing is then ordered based on NAAT results and/or point-of-care urethral smear for *Neisseria gonorrhoeae*. In case of detection of an STI, partner notification is necessary to prevent re-infection, preventing potentially significant morbidity, and prevent onward transmission (9, 15). Moreover, in those suffering from one STI, testing for relevant serologically detectable STIs can be considered, including Hepatitis B and C, syphilis, and HIV (6).

## Gonococcal urethritis

### Neisseria gonorrhoeae

Gonorrhea is the second most common bacterial STI in Europe with an increase in the incidence of several hundred percent in the last 5 years (12, 16). The causative agent *Neisseria gonorrhoeae* is a Gram-negative bacteria that can be transmitted by unprotected sexual intercourse or direct inoculation of the epithelial mucosa *via* other routes. Infections with this pathogen typically have a short incubation period (1–10 days) (11, 12, 17). It is believed that up to 20% of all urethritis cases are caused by NG (12). An infection confers no immunity. Antimicrobial resistance (AMR) in gonorrhea is a global public health concern, as NG offers all main variants of resistance mechanisms and multi-drug resistant gonorrhea cases have been reported [(17) MolBiol]. Several AMR determinants have been described for all relevant antibiotics, including ceftriaxone, cefixime, azithromycin, spectinomycin, ciprofloxacin, or ofloxacin [(17) MolBiol]. The World Health Organization (WHO) offers a gonococcal antimicrobial surveillance program (GASP) since 1992. In their latest retrospective analysis, resistance rates ranging from 0 to 22% (cephalosporins), 0 to 60% (azithromycin), and 0 to 100% (ciprofloxacin) were reported from all over the world (18). Globally, resistance to ciprofloxacin remains very high, ranging from 49% (European region) to 93% (South East Asia). In general, ciprofloxacin should be only administered in those NG infections that are known to be susceptible (19).

### Clinical symptoms

Almost 90% of infected men develop symptoms, mainly discharge (>80%), followed by dysuria (12, 19). In severe or prolonged disease, ascending infections are possible, and might end in prostatitis, epididymitis, or epididymo-orchitis (12, 19, 20). Gonococcal septicemia or disseminated gonococcal infection (DGI) can occur, resulting in clinical symptoms of petechiae or pustules at the acral areas, tenosynovitis, poly-arthralgia, or septic arthritis (6).

### Diagnostic methods

Detection of *NG* can be performed microscopically (with a specificity of up to 99% and sensitivity of up to 95% in symptomatic men) due to the presence of intracellular Gram-negative diplococci within PMNLs at a Methylene blue or Gram stain, *via* culture (which is much more important for the inevitable susceptibility testing) or by direct detection using NAATs (6, 11, 12, 17). Recommended specimens are urethral smears or first void urine. With the latter, the first 15–30 ml of first void urine shall be collected after micturition abstinence for at least 60 min (11, 12). Culture of this microorganism is usually needed for antibiotic susceptibility testing (11–13, 19). Undoubtful, NAATs are the recommended state-of-the-art testing for gonorrhea (see Table 1) (12, 13, 19).

**TABLE 1:** Recommended testing methods for most relevant sexually transmitted urethritis pathogens.

Pathogen/specimen type	Microscopy	Culture	NAATs
*Neisseria gonorrhoeae*	After staining with methylene blue or Gram stain	Inevitable for antimicrobial resistance testing	Recommended test of choice

First void urine	−	−	+

Urethral swab	+	+	+

Serology	Not recommended		

*Chlamydia trachomatis*	Intracellular bacterium, impossible	Only if NAATs are not available	Recommended test of choice

First void urine	−	−	+

Urethral swab	−	±	+

Serology	Not recommended as only invasive CT infections might lead to detectable levels of antibodies		

*Mycoplasma genitalium*	No cell wall, impossible	Slow growing, would take 6 months	Only recommended test of choice

First void urine	−	−	+

Urethral swab	−	−	+

Serology	Not recommended, cross reactivity		

In men in whom Gram-negative diplococci are observed by Gram stain smear that test negative for NG by NAAT, *N. meningitidis* clonal complex 11 strains should be considered. Recent studies, primarily from the United States, have shown that these bacteria may be an important cause of urethritis in healthy men, and can sometimes disseminate and cause fatal disseminated disease in immunocompromised individuals (6, 14).

### Treatment

The European section of the International Union Against Sexually Transmitted Infections (IUSTI) recommends – in case of unknown antimicrobial susceptibility – ceftriaxone 1 g plus azithromycin 2 g as single doses OR ceftriaxone 1 g monotherapy as a single dose (12). The CDC recommends ceftriaxone 500 mg i.m. for those weighing <150 kg, and 1 g for those above (6). Treatment for *Neisseria meningitidis* follows the same regimen (6).

According to the European IUSTI guidelines tests for other STIs and sexual abstinence for 7 days after treatment are recommended (12, 17). In the case of ceftriaxone monotherapy or alternative treatment regimens, a TOC is mandatory according to the European IUSTI guidelines (12). There is disagreement concerning whether TOC is necessary for Europe due to the low frequency of ceftriaxone resistance. According to the CDC, a TOC for those treated with a recommended regimen for oro-ano-genital gonorrhea is not necessary (6). All sexual partners of the last 60 days to 3 months shall be informed (6, 12).

## Non-gonococcal urethritis

**FIGURE 1:**
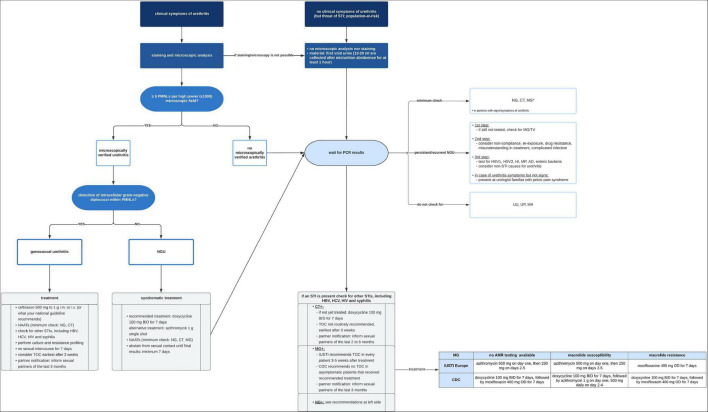
Recommended diagnostic and therapeutic algorithm in case of urethritis or risk of urethral STI. AD, Adenovirus; BID, bis in die (twice a day); CDC, center of disease control and prevention; CT, *Chlamydia trachomatis;* HBV, hepatitis B virus; HCV, hepatitis C virus; HI, *Haemophilus influenzae*; HIV, human immunodeficiency virus; HSV1, herpes simplex virus 1; HSV2, herpes simplex virus *2*; i.m., intramuscular; IUSTI, European section of the international union against sexually transmitted infections; i.v., intravenous; MG, *Mycoplasma genitalium*; MH, *Mycoplasma hominis*; MP, *Mycoplasma penetrans*; NAATs, nucleic acid amplification tests; NG, *Neisseria gonorrhoeae*; PMNLs, polymorphonuclear leukocytes; p.o., *per os*; TOC, test of cure; TV, *Trichomonas vaginalis*; UP, Ureaplasma parvum; UU, *Ureaplasma urealyticum.*

Non-gonococcal urethritis is mainly caused by *Chlamydia trachomatis* (up to 50%), followed by *Mycoplasma genitalium*, which is believed to cause 15–50%, and *Trichomonas vaginalis* (1–20%), depending on the prevalence in the respective countries (1–6, 8). Less common pathogens include Herpes simplex virus 1, Herpes simplex virus 2, Adenovirus, *or Trichomonas vaginalis* (7, 8). Several new pathogens have been implicated in NGU recently, including *Haemophilus influenzae* (HI) and *Mycoplasma penetrans* (MP) (8). Still, no pathogen was identified in up to 50% of men with NGU in some recent studies (1, 2, 7, 8). In the case of typical symptoms and microscopically verified urethritis, syndromically based treatment shall be started (see Figure 1) (1, 6). The first line recommended treatment regimen is doxycycline 100 mg BID for 7 days and as an alternative azithromycin 1 g p.o. as a single dose (6).

Only recently Toh et al. (21) evaluated various cure outcomes (clinical, Gram stain, microbiological) in men who received azithromycin for NGU. They observed that clinical cure (loss of symptoms) predicted microbiological cure (Test of cure *via* NAATs) for those infected with CT, UU, and TV. However, that was not the case for MG, as microbiological failure has been observed in men who experienced clinical cures (21). Macrolide-resistant MG infections were highly linked to microbiological failure (21). The authors, therefore, concluded that in azithromycin-treated MG-NGU infected men, the microbiological cure must be gained, at least in those settings where macrolide resistance cannot be tested (21). Interestingly, until today, no one has investigated NGU outcomes according to the new CDC recommendations in the United States.

### Recurrent/persisting non-gonococcal urethritis

In case of recurrent (symptoms recurring within 30–90 days after treatment, prevalence of up to 20%) or persisting (persisting symptoms despite treatment, prevalence up to 25%) NGU (2) non-compliance, re-exposure, drug resistance, misunderstanding in treatment, or complicated infection must be borne in mind (1, 2, 6). If not done previously, MG testing is inevitable in this situation (6).

### Chlamydia trachomatis

*Chlamydia trachomatis* is the most common bacterial STI and cause of urethritis in both Europe and the United States (6, 13, 22). CT is an obligate intracellular bacterial pathogen that only replicates in living human epithelial mucosa (13, 22–24). To date, 12 relevant uro-ano-genital serovars are known. Serovars D to K cause classical urogenital STIs and three variants (L1, L2, and L3) can cause a more infrequently detected STI called lymphogranuloma venereum (LGV) (22, 23). CT is transmitted *via* unprotected sexual intercourse and has an incubation period ranging from 1 to 4 weeks (9, 22, 24). It is unclear if a prior infection confers protective immunity, however, partial immunity is likely.

### Clinical symptoms

Some studies have observed asymptomatic courses in approximately 50% of men (22). However, other studies have observed that urethral inflammation can be detected in most men with CT, and in a case–control study by Jordan et al. where symptoms and inflammation were monitored in the cases and controls, extremely low rates of silent CT infections were observed (7). In symptomatic men, dysuria and clear or whitish discharge are common (24). Collection of an adequate and detailed history is important, as many men might not report minor symptoms otherwise. In the case of ascending infection, epididymitis and orchitis are rare but serious sequelae (22–24).

### Diagnostic methods

Urethritis can be diagnosed *via* a Gram stain. As CT cannot easily and/or consistently be visualized in urethral specimens, NAATs are the recommended choice for verification and are believed to be positive within the first 3–14 days after the sexual intercourse (6, 22, 24). Culture is possible, but not routinely recommended, as it is technically challenging, time-consuming, and less sensitive than NAATs (22, 24). Conversely, NAATs are more likely to yield false positives due to residual DNA, and TOC *via* NAAT shall not be performed earlier than 3 weeks after ending the treatment. Suitable samples are urethral smears or first void urine (see Table 1) (6).

### Treatment

The European IUSTI guideline recommends first-line treatment as doxycycline 100 mg two times a day for 1 week or second-line treatment with azithromycin 1 g p.o. as a single dose (22). The CDC now recommends doxycycline as the first-line regimen as well, because azithromycin is inferior for the elimination of concomitant rectal CT infections (6, 25).

All partners of the last 2–6 months shall be informed (6, 22). In case of complicated CT infection, treatment duration should be extended to 2 weeks. Those infected should abstain from sexual intercourse for 1 week after they and their partners completed treatment and symptoms fade (6, 22). If the recommended treatment was prescribed, a TOC is not routinely recommended but can be performed either 3–4 weeks or 3 months after treatment (6, 22). CDC guidelines recommend the 3-month follow-up visit for re-testing (6).

### Mycoplasma genitalium

*Mycoplasma genitalium* is an atypical small parasitic bacterium. Infections with this pathogen have an incubation period of approximately 4–8 weeks (9, 26, 27). Transmission occurs *via* unprotected sexual intercourse (27). After decades of neglect, it has recently become clear that MG is an important cause of urethritis, and especially treatment-refractory urethritis, and possibly other syndromes including epididymo-orchitis and proctitis, MG prevalence can be as high as 4% in the general population (9, 26, 27). The prevalence of MG in NGU, however, ranges from 10 to 35% (27). The relevance of asymptomatic MG infections remains unclear (6).

Due to the lack of a cell wall, only a few antibiotics have activity against mycoplasmas including tetracyclines, macrolides, streptogramins (disrupting protein synthesis), and fluoroquinolones (disrupting DNA synthesis) (27–28). Antimicrobial resistance of these microorganisms is growing, and consecutive treatment failures with both macrolides and fluoroquinolones have been observed (28, 29). Macrolide resistance has been rapidly increasing, which is believed to range from <4% (Russia) to 69% (Japan), and MG has been called an emerging public health issue (6, 26, 28–30). In the case of fluoroquinolones, resistance rates are lower (8–20%) and seem to be more consistent around the globe (28, 29). Already in 2017, a study by Murray et al. observed an increasing incidence of macrolide- and fluoroquinolone-resistant *MG* in Australia. They detected a high frequency of ParC S83 changes linked to moxifloxacin failure and 10% of their MG isolates were predicted to be resistant to macrolides and fluoroquinolones (31). Some patients with these isolates respond positively to pristinamycin (31).

Due to the increase in resistance, other treatment options must be explored. One of those is sitafloxacin, a fluoroquinolone (30). In a study by Murray et al., the authors tried to identify mutations responsible for the failure of fluoroquinolones. The results indicated that mutations of *parC* G248T/S83I are associated with failure of both moxifloxacin and sitafloxacin and recommend paying attention to their results concerning the development of next-generation resistance assays (30).

Untreatable MG is already a reality, and due to that threat, inevitably, resistance-guided treatment strategies must be followed, ideally including surveillance on MG AMR (27, 28, 30).

### Clinical symptoms

Many MG infections run an asymptomatic course. In symptomatic men, urethral discharge or dysuria are the most common symptoms (27).

### Diagnostic methods

The preferred diagnostic methods are NAATs (a temporal gap of at least 2 weeks after exposure is recommended) (9, 27). Testing is highly recommended in symptomatic patients suffering from symptoms or signs of urethritis, recurrent and/or persistent NGU, dysuria with no known other etiology, proctitis (after NG and CT were excluded), or if it is a male patient younger than 50 years suffering from acute epididymo-orchitis (6, 26, 27).

First void urine and (less recommended due to invasiveness) urethral swabs are suitable (see Table 1) (27).

### Treatment

The European IUSTI guideline recommends (in lack of resistance testing OR macrolide susceptibility) either azithromycin 500 mg on day 1, followed by 250 mg on days 2–5, or Josamycine 500 mg three times daily for 10 days (9, 27). In the case of macrolide resistance, moxifloxacin 400 mg for 7 days is recommended (27). In case of complicated MG infection (e.g., epididymitis), moxifloxacin 400 mg for 14 days is recommended (27).

The Centers for Disease Control recommendations are based on the high macrolide resistance rates in the United States and follow a two-stage approach (6). If AMR testing is not available OR MG is macrolide resistant, treatment shall be performed with doxycycline 100 mg BID for 7 days followed by a course of moxifloxacin 400 mg one time daily for another week (6). The idea behind this regimen is that using doxycycline for decreasing bacterial load and increasing the likelihood of moxifloxacin will be effective (6). If the MG isolate is macrolide sensitive, treatment is recommended with doxycycline 100 mg BID for 7 days, followed by azithromycin 1 g orally on day 1, and 500 mg daily on days 2–4 (6).

Resistance-guided sequential therapy has shown great cure rates, as shown by a study published by Durukan et al. (29). They evaluated the efficacy and tolerability of doxycycline–moxifloxacin and doxycycline–2.5 g azithromycin (29). In the case of NGU, patients received doxycycline 100 mg BID for 7 days. In case of MG infection, macrolide-susceptible cases received 1 g azithromycin, followed by 500 mg daily for 3 days. Macrolide-resistant cases received 400 mg moxifloxacin for 7 days. A TOC was recommended 2–3 weeks after treatment (29). Both cure rates were above 92%, with excellent tolerability (29). Resistance-guided sequential therapy has been incorporated by the Australians and modified in the United Kingdom guidelines. In the case of NGU, doxycycline (100 mg BID, 7 days) is prescribed as a first step. If this fails, subsequent treatment with azithromycin 1 g orally on day 1, and 500 mg daily on days 2–4 OR moxifloxacin for 7–10 days is recommended (27).

Men who are infected with MG should abstain from sexual intercourse until after they (and their sexual partners) complete treatment, have no more symptoms, and have a negative TOC (3–5 weeks after the end of treatment) (27). In the case of azithromycin-treated MG–NGU cases, Toh et al. observed that 1-week abstinence seems to be insufficient and recommended a test of cure be performed in (probably) all MG-infected patients (21). They, moreover, recommend abstinence from unprotected sex until a test of cure, to avoid MG transmission (21). CDC does not recommend TOC in those who are asymptomatic and received a recommended CDC treatment regimen (6). However, because these recommendations are relatively new, it may be necessary to revisit these recommendations when more follow-up data are available.

## Other non-gonococcal urethritis-causative pathogens

In addition to the above-mentioned pathogens, several other microorganisms may cause urethritis. Among those are Herpes simplex viruses 1 and 2, *Trichomonas vaginalis*, Adenovirus, *Treponema pallidum*, *Haemophilus* species, and *M. penetrans* (6).

### Herpes simplex virus 1 and herpes simplex virus 2

The highly prevalent DNA viruses are HSV-1 and –2, which may cause NGU and explain 2–10% of NGU cases (32, 33). Only one-third of patients present with herpetiform lesions on the skin itself, the majority suffer from meatitis and dysuria but without discharge (32). Constitutional symptoms are common (32). Transmission is most likely *via* insertive oral sex (33). Diagnosis of HSV–NGU is only possible *via* NAATs using urethral swabs or first void urine, with high sensitivity (6). Most cases do not need specific (antiviral) treatment.

### Adenovirus

Adenovirus is a contagious DNA virus with seven serotypes (A-G), causing mainly respiratory tract infection, conjunctivitis, and more seldom urethritis (2–4% of NGU cases; serovar D seems to be the most common type) among some others (34). Transmission occurs most likely *via* unprotected oral sexual intercourse within the last month (33, 34). A typical clinical presentation consists of mainly meatitis, dysuria, clear or mucoid discharge, and (mostly bilateral) conjunctivitis (33, 34). Diagnosis might be made clinically, in case of the presence of the triad of meatitis–urethritis–conjunctivitis, especially from September to March. For academic interests, NAATs can be performed on urethral and conjunctival swabs (33, 34). Specific treatment is unnecessary, in this self-limiting disease. Patients should abstain from sexual intercourse until the symptoms are resolved.

### Trichomonas vaginalis

*Trichomonas vaginalis*, accounts worldwide for the most common parasitic sexually transmitted infection, called trichomoniasis with an incidence of several hundreds of millions annually, but is uncommon in the European population and rare in the United States population, affecting only 0.5% of men in the United States; having only higher rates in those incarcerated (prevalence: up to 8%) (6, 35). TV is a flagellated, extracellular protozoan that can infect urogenital squamous epithelia (35–37). Its incubation period ranges from some days to 1 month (35). Transmission occurs *via* unprotected sexual intercourse or direct mucosal contact (35). Men seldom have symptoms, however, signs of urethritis, epididymitis, or prostatitis are possible (6, 35, 36). NAATs are the recommended test of choice. Urine is the preferred material of symptomatic individuals (35, 36). Microscopically, the protozoan can be detected if microscopy is performed rapidly, but as it has a low sensitivity (∼ 50%) (6, 35, 36). While Kissinger et al. and Van Gerwen et al. do not recommend culturing in men, according to the CDC multiple specimens for inoculation of one culture can be used (6).

According to the European IUSTI guideline, metronidazole 400–500 mg BID for 5–7 days (with simultaneous abstinence of alcohol consumption for at least 24 h after the end of treatment) is recommended (37). According to the CDC, men should be treated with a single dosage of metronidazole 2 g orally as a single dose; however, no valid data from meta-analysis are available for the treatment of men (6). Those who are infected shall abstain from sexual intercourse after they and their partners complete treatment and symptoms have resolved (6, 35, 37). A TOC is not necessary for asymptomatic patients (37). Current partners should receive presumptive therapy [Workwoski].

### Haemophilus influenzae

*Haemophilus influenzae* (HI) is a fastidious bacterial pathogen that usually colonizes the upper respiratory tract, but may also be transmitted to the urethra *via* oral sex (6, 8). The prevalence of HI–NGU ranges from 7.4 to 14% (8, 38). Diagnosis of HI has been performed *via* serotyping and culturing or NAATs (8, 38). According to the study by Ito in 2017, HI was successfully treated by recommended Japanese approach, but there have been no large follow-up studies concerning response to the contemporary European and United States NGU regimens (38). However, infections with most of all the above-mentioned rare organisms are either self-limited, respond to existing NGU management regimens, or are simply too rare to be clinically relevant.

### *Mycoplasma hominis, Ureaplasma urealyticum, Ureaplasma parvum*, and *Mycoplasma penetrans*

In 2018, a position statement from the European STI Guidelines Editorial Board was published, recommending that diagnostic laboratories abstain from testing *Mycoplasma hominis, Ureaplasma urealyticum*, and *Ureaplasma parvum*, as urethral colonization with these microorganisms, without the development of disease is common (39). *Ureaplasma urealyticum (UU*) has been discussed as a causative pathogen of NGU but was considered to be relevant only in the case of high bacterial load (6, 21, 39). However, Jordan et al. (7) performed a case–control study to identify the risk of UU in NGU cases. They found that UU–mono-infection is not associated with non-gonococcal urethritis. UU seems to colonize the urethra, without causing inflammation, and therefore UU is more and more considered as a non-NGU pathogen (7, 8, 21). Interestingly Jordan et al. observed frequent co-existence with other NGU pathogens (7).

*Mycoplasma penetrans* is a Gram-positive intracellular bacterium found in the urogenital or respiratory tract. MP accounted for 21% of previously idiopathic NGU cases in a case–control study by Srinivasan et al. (8). Interestingly more men who have sex with men (MSM) suffered from MP–urethritis compared to men who have sex with women (MSW) and showed an association of MP–urethritis only in the MSM group (8). Detection of MP is possible *via* the usage of NAATs (8). MP was not included in the most recent CDC or IUSTI treatment recommendations and these should be investigated in future trials (8), but unpublished clinical observations indicate that most of these infections respond well to moxifloxacin but not macrolides (DE Nelson, personal communication).

## Persistent symptoms of urethritis

If urethritis and/or symptoms persist, individuals should be re-tested for MG and TV. Rarer STIs and STI-independent causes of urethral inflammation should also be considered. In those reporting symptoms, but lack signs of urethritis, presentation to a urologist, familiar with pelvic pain syndrome should be recommended (6).

## Conclusion and future perspectives

Although great success was made in the diagnosis and treatment of STIs in the last decades, the incidence of many STIs is increasing for a variety of reasons. An ongoing threat is the rapid emergence of antimicrobial resistance of NG to a multitude of classes of antibiotics (16), and an even more significant problem is untreatable MG infections, which is already a reality (28). Venereologists are in great need of sufficient vaccines for preventing/treating gonorrhea and the main denominator is the lack of clinical trials so far; however, preclinical trials are ongoing, handling that topic (16). Especially adolescents and young adults (“AYA”) account for a large number of those infected with STI and therefore, infections with NG, CT, and MG are a major concern for sexual and reproductive health globally (40). Especially, infections with CT are a major topic, as many follow an asymptomatic course and acquire the infection most likely during their reproductive phase in a lifetime (40). Educational programs for the group of AYA and those with risk behavior are essential. In most countries, individuals are not aware of the specialty of venereology and present themselves to general practitioners or urologists first. Those, however, might not be familiar with the latest developments in STIs. Therefore, diagnosis and correct treatment might be delayed, resulting in significant morbidity and a complicated course of the infection. This review gives a comprehensive overview for all physicians, of the most common reason for presentation with urethritis-like symptoms and the authors present a proposal of a diagnostic and therapeutic algorithm that can be used in clinical routine.

## Author contributions

BS: conceptualization, resources, writing – original draft and review and editing, and visualization. BK and PK: writing – review and editing and supervision. GH: supervision, resources, and writing – review and editing. All authors contributed to the article and approved the submitted version.
